# Drug-Induced Pemphigus Vulgaris Following Ceftriaxone Use

**DOI:** 10.7759/cureus.110369

**Published:** 2026-06-06

**Authors:** Alpha Amadi, Nicholette Murray-Bruce, Jovan Gayle, Perkins Mukunyadzi, John Mark P Pabona

**Affiliations:** 1 Department of Internal Medicine, Catholic Health Initiatives (CHI) St. Vincent, Hot Springs, USA; 2 Department of Pulmonary and Critical Care Medicine, Catholic Health Initiatives (CHI) St. Vincent, Hot Springs, USA; 3 Department of Pathology and Laboratory Medicine, Catholic Health Initiatives (CHI) St. Vincent, Hot Springs, USA

**Keywords:** adverse side effect, atypical pemphigus vulgaris, autoimmune, ceftriaxone therapy, drug-induced pemphigus, flaccid bulla, iv ceftriaxone, third-generation cephalosporins

## Abstract

Pemphigus vulgaris (PV) is a rare, potentially life-threatening autoimmune blistering disorder characterized by intraepidermal acantholysis caused by IgG autoantibodies directed against desmoglein 1 and 3. Although most cases are idiopathic, certain medications, including antibiotics, have been implicated as potential triggers in susceptible individuals. We report the case of a 61-year-old man with a history of nonischemic cardiomyopathy, chronic kidney disease, and substance use disorder who developed widespread flaccid bullae six days after initiation of intravenous ceftriaxone for a suspected urinary tract infection. Histopathologic examination demonstrated suprabasal intraepidermal acantholysis with intraepidermal vesicles containing neutrophils and eosinophils, along with preservation of the basal epidermal layer in a characteristic “row of tombstones” pattern, findings highly suggestive of PV. Ceftriaxone was promptly discontinued, and the patient was treated with high-dose systemic corticosteroids, resulting in marked clinical improvement. Ceftriaxone-induced PV is an exceedingly rare adverse reaction, with few cases reported in the literature. The proposed pathogenesis involves drug-induced neoantigen formation or stimulation of pathogenic autoantibody production in genetically predisposed individuals. Early recognition of drug-induced PV is essential, as continued exposure may result in extensive mucocutaneous involvement, secondary infection, and increased morbidity. Prompt withdrawal of the offending agent and initiation of immunosuppressive therapy are critical for improving clinical outcomes.

## Introduction

Pemphigus vulgaris (PV) is an uncommon and potentially fatal autoimmune blistering condition defined by intraepidermal acantholysis driven by autoantibodies targeting desmoglein 1 ± 3 [1]. The disease manifests clinically as flaccid blisters and erosions of the skin and mucous membranes. Without appropriate treatment, PV can lead to life-threatening sequelae including secondary infection, sepsis, and significant fluid losses [1]. While the pathogenesis of PV is considered multifactorial, numerous pharmacologic agents have been identified as potential triggers, among them penicillamine, angiotensin-converting enzyme (ACE) inhibitors, nonsteroidal anti-inflammatory drugs (NSAIDs), imiquimod, and select cephalosporins such as cephalexin, cefadroxil, ceftazidime, and, on rare occasions, cefixime [2-6].

Ceftriaxone is a third-generation cephalosporin employed extensively for both empiric and directed therapy of Gram-positive and Gram-negative infections. Although it is recognized for its favorable efficacy and safety profile, cutaneous adverse effects-though generally mild and infrequent-have been documented [7]. Drug-induced pemphigus constitutes a severe immune-mediated reaction that has only rarely been linked to cephalosporin use [4]. Thiol- and phenol-containing drugs are the agents most frequently implicated in triggering pemphigus, as they can directly disrupt keratinocyte membrane integrity; in contrast, non-thiol/non-phenol agents such as ACE inhibitors, NSAIDs, and calcium channel blockers tend to provoke the PV variant rather than pemphigus foliaceus [8].

We describe a case of probable ceftriaxone-induced PV in a patient hospitalized following ventricular fibrillation cardiac arrest. This report contributes to the expanding body of evidence regarding cephalosporin-associated hypersensitivity reactions.

## Case presentation

A 61-year-old man with a history of hypertension, stage 3B chronic kidney disease, nonischemic dilated cardiomyopathy with previously improved ejection fraction (50%-55%), chronic obstructive pulmonary disease, gout, and substance use disorder was transferred to our tertiary care center after recurrent automated implantable cardioverter-defibrillator (AICD) shocks. His home medications included lisinopril, metoprolol, spironolactone, colchicine, allopurinol, inhaled corticosteroids, a long-acting muscarinic antagonist, and a long-acting beta-agonist.

Prior to transfer, he experienced eight AICD discharges at home and was found by emergency medical services to be in ventricular fibrillation, requiring four external cardioversions and two 150 mg intravenous amiodarone boluses. At the referring hospital, he was agitated and dyspneic, requiring endotracheal intubation; he subsequently converted to normal sinus rhythm prior to transfer. On arrival, he remained intubated and sedated on amiodarone, norepinephrine, and propofol infusions.

Laboratory evaluation demonstrated leukocytosis with left shift, elevated creatine kinase and troponin, and acute kidney injury on chronic kidney disease (Table 1). Urinalysis suggested a urinary tract infection, and blood and urine cultures were obtained. Urine toxicology was positive for amphetamines. Chest radiograph, CT head, and CT cervical spine were unremarkable.

**Table 1:** Laboratory evaluation WBC: white blood cell; RBC: red blood cell; GFR: glomerular filtration rate; NT: not tested; H: high; L: low

Test	Day 1	Day 6	Normal range
WBC (K/uL)	19.1 (H)	14.7 (H)	4.5-11.0
Neutrophils (%)	84 (H)	75	42-75
Eosinophils (%)	0	5	0-5
RBC (M/uL)	5.25	5.46	4.50-5.90
Hemoglobin (g/dL)	15.4	15.2	13.5-17.5
Platelet (K/uL)	231	223	150-400
Creatine Kinase (U/L)	2967 (H)	NT	30-200
Troponin (pg/mL)	2291 (H)	NT	<36
Sodium (mmol/L)	137	144	136-145
Potassium (mmol/L)	4.7	4.3	3.5-5.1
Chloride (mmol/L)	96	106	98-107
Bicarbonate (mmol/L)	29	28	22-29
Blood urea nitrogen (mg/dL)	66 (H)	53 (H)	7-26
Creatinine (mg/dL)	3.76 (H)	2.32 (H)	0.72-1.25
GFR (mL/minute/1.73 m²)	17 (L)	31 (L)	>60

Hospital course

The patient was managed for acute hypoxemic and hypercapnic chronic respiratory failure requiring mechanical ventilation, non-ST-elevation myocardial infarction (NSTEMI), postventricular fibrillation cardiac arrest, rhabdomyolysis likely secondary to methamphetamine use, acute kidney injury on chronic kidney disease, suspected urinary tract infection, and acute metabolic encephalopathy.

Empiric therapy with ceftriaxone (1 g daily) and azithromycin (500 mg daily) was initiated for suspected urinary tract infection and possible aspiration pneumonia. A heparin infusion was started for NSTEMI. Cardiology was consulted for recurrent arrhythmia and NSTEMI management.

On hospital day 2, azithromycin was discontinued due to a brief run of ventricular tachycardia and electrolyte abnormalities, which were corrected. On day 3, left heart catheterization demonstrated no significant coronary artery disease, consistent with nonischemic dilated cardiomyopathy.

**Figure 1:**
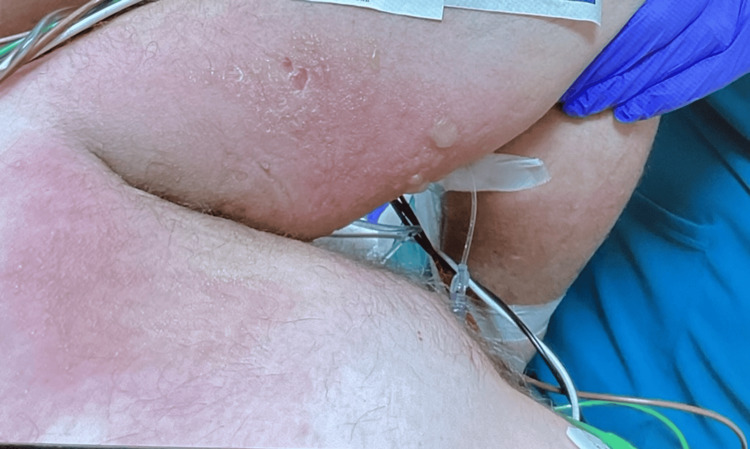
Dermatologic presentation of the clinical image demonstrating flaccid superficial bullae over the posterior right upper thoracic region, right upper arm, and axilla, with ruptured erythematous blisters and serous fluid-filled bullae on day 6 of presentation, findings consistent with an intraepidermal blistering disorder

**Figure 2:**
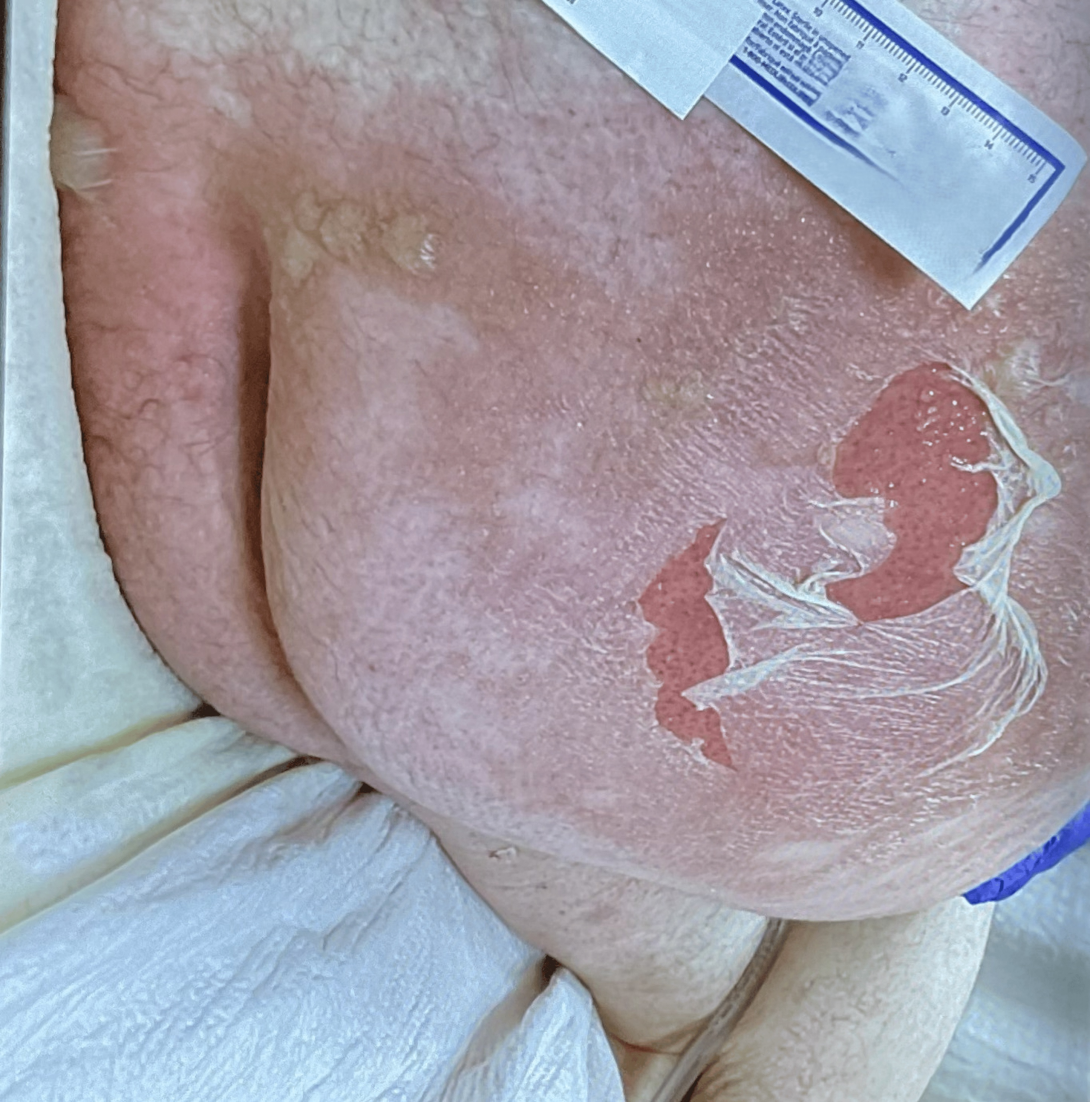
Dermatologic presentation of the clinical image demonstrating flaccid superficial bullae and postbullous erosions involving the gluteal region on an erythematous base, with multiple serous fluid-filled bullae, findings consistent with an intraepidermal blistering disorder on day 6 of presentation

On day 5, erythematous skin changes were noted over the trunk and upper extremities and were monitored. On day 6, the patient was extubated but developed respiratory distress within hours, requiring reintubation. Later that day, the erythematous lesions progressed to flaccid bullae with a positive Nikolsky sign, involving the back, bilateral axillae, upper arms, and bilateral gluteal regions (Figures 1, 2), affecting an estimated 45% of the total body surface area. Initial mucosal examination was limited in the setting of critical illness and sedation; however, no oral lesions were identified on formal examination once the diagnosis was suspected.

Wound care was consulted, and a skin biopsy was obtained. Given concern for a drug-induced process, ceftriaxone was discontinued, and doxycycline (100 mg twice daily) was initiated as an alternative antibiotic. High-dose intravenous methylprednisolone (80 mg every eight hours) was started. The amiodarone infusion was transitioned to oral amiodarone (200 mg twice daily) via orogastric tube.

On day 8, the patient was successfully extubated and remained hemodynamically stable. He was alert and oriented, able to recall events surrounding his cardiac arrest, and reported significant discomfort from the cutaneous lesions. Leukocytosis and renal function improved.

**Figure 3:**
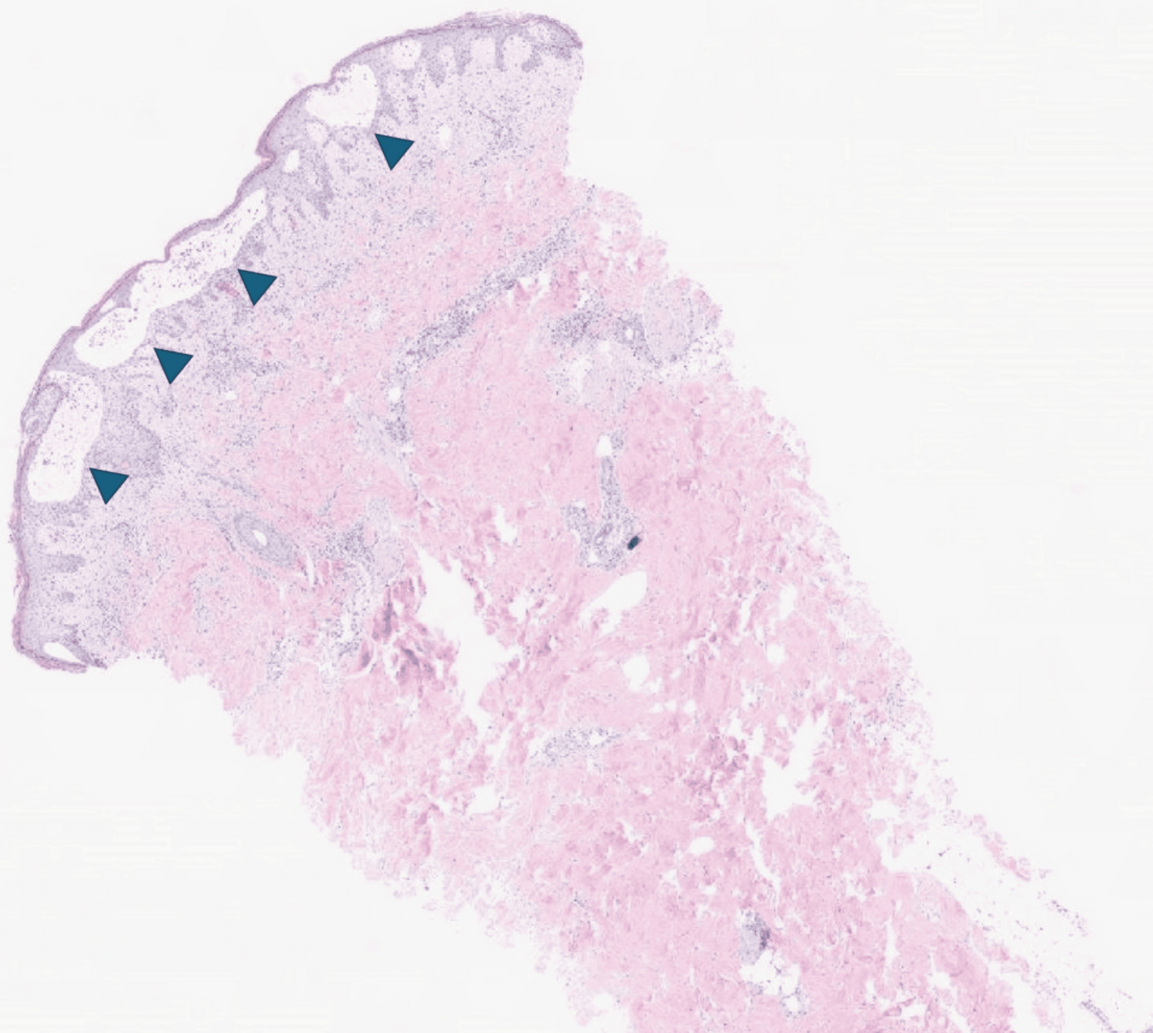
10× magnification of skin biopsy Hematoxylin and eosin-stained section of skin biopsy demonstrating prominent intraepidermal acantholysis (arrows), consistent with a suprabasal blistering process

**Figure 4:**
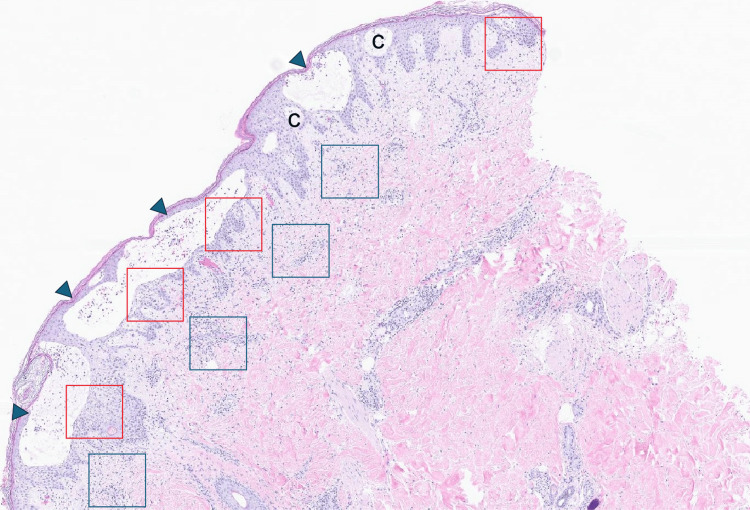
20× magnification of skin biopsy Hematoxylin and eosin-stained skin biopsy showing intraepidermal vesicles with acantholysis (arrows), epidermal neutrophilic and eosinophilic infiltrates (red box), and a characteristic “row of tombstones” pattern at the base of the cleft with dermal inflammatory infiltrates (blue box). Additional areas of acantholysis and blister cavity formation (C) are present

**Figure 5:**
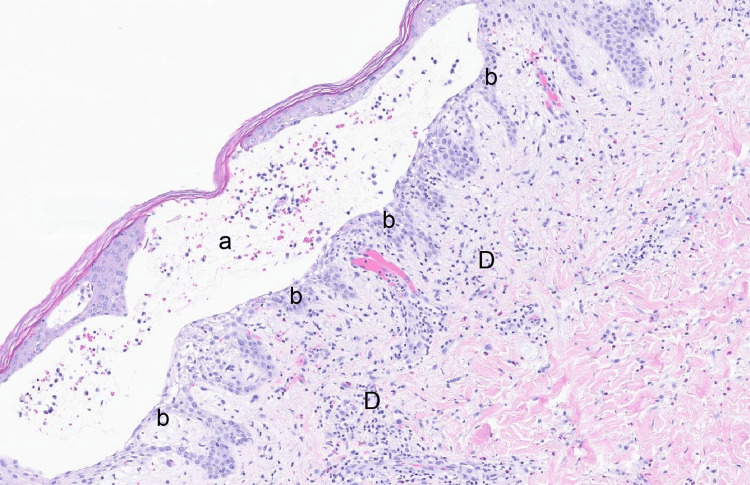
40× magnification of skin biopsy Hematoxylin and eosin-stained skin biopsy showing intravesicular mononuclear inflammatory infiltrates (a), suprabasal intraepidermal clefting with a preserved basal epidermal layer (b), and dermal inflammatory infiltrates (D). Full-thickness epidermal necrosis and scattered necrotic keratinocytes are absent

By day 10, cutaneous lesions showed marked improvement. Histopathologic examination revealed suprabasal intraepidermal acantholysis with intraepidermal vesicles containing occasional neutrophils and eosinophils. The dermis demonstrated a mixed inflammatory infiltrate composed of neutrophils and eosinophils (Figures 3-5). These findings were most consistent with PV in the setting of a suspected drug-induced autoimmune blistering disorder. Given the limited biopsy specimen and the patient’s clinical improvement with drug discontinuation and systemic corticosteroids, repeat biopsy and direct immunofluorescence testing were not performed. The patient continued to improve with corticosteroid therapy and was discharged on hospital day 11 with planned outpatient follow-up; however, he was subsequently lost to follow-up.

## Discussion

Cephalosporins exert their bactericidal effect by inhibiting cell wall synthesis through inactivation of penicillin-binding proteins, which are critical for peptidoglycan cross-linking; disruption of this process ultimately results in bacterial cell lysis. Ceftriaxone, as a third-generation agent, maintains broad-spectrum activity against both Gram-positive and Gram-negative organisms and demonstrates considerable stability against many β-lactamases, including penicillinases and cephalosporinases [9]. It is frequently prescribed for urinary tract infections and a range of other bacterial infections [9]. Notably, ceftriaxone is among the few antibiotics, and the only cephalosporin, with dependable penetration across the blood-brain barrier, establishing it as a first-line option for bacterial meningitis [10].

Ceftriaxone is generally well tolerated; however, recognized adverse effects encompass allergic reactions secondary to immune activation, bone marrow suppression or immune-mediated cytopenias, biliary and renal precipitation (particularly in patients receiving calcium-containing intravenous fluids or those with predisposing risk factors), central nervous system toxicity, diarrhea, and hepatic enzyme elevations. Severe cutaneous reactions including Stevens-Johnson syndrome (SJS) and toxic epidermal necrolysis (TEN) have been reported but remain rare [9].

PV is an autoimmune blistering disorder in which flaccid bullae develop on the skin and mucosal surfaces. The disease results from a combination of genetic predisposition and environmental triggers that lead to the generation of pathogenic IgG autoantibodies against desmoglein 3, with or without concurrent targeting of desmoglein 1, both of which are critical adhesion molecules within the epidermis and mucosal epithelium [1,3]. The consequent loss of intercellular adhesion among keratinocytes produces acantholysis and intraepidermal blister formation, often accompanied by a positive Nikolsky sign. Although oral mucosal involvement is a hallmark of PV, no mucosal lesions were detected on formal examination in the present case. PV is classified as a type II hypersensitivity reaction and is observed most frequently in older adults [1,8]. Diagnosis depends on early clinical suspicion combined with skin biopsy for histopathological assessment and immunofluorescence studies.

On histopathology, PV characteristically demonstrates suprabasal acantholysis with a "row of tombstones" pattern along the basal layer adjacent to the suprabasal cleft. The underlying dermis may show a mild inflammatory infiltrate composed primarily of lymphocytes, occasionally accompanied by eosinophils or neutrophils. Direct immunofluorescence typically reveals intercellular IgG deposition in a reticular (fishnet) pattern throughout the epidermis. Serologic testing, including indirect immunofluorescence and enzyme-linked immunosorbent assay, commonly identifies circulating IgG autoantibodies directed against desmoglein 3 ± desmoglein 1 [1].

The estimated incidence of PV is approximately one per 100,000 persons. Although its etiology is multifactorial, drug-induced pemphigus is a well-recognized precipitant, with PV being the most common clinical variant; drug-induced pemphigus occurs at an estimated rate of 0.1-0.5 per 100,000 individuals per year [8].

Ceftriaxone-induced PV is an exceedingly rare adverse event, with only isolated cases documented in the literature [11]. PV is associated with several human leukocyte antigen (HLA) class II alleles, most notably DQB105:03 and DRB104:02 [1]. Interestingly, low-titer antidesmoglein autoantibodies have been detected in roughly half of clinically unaffected first-degree relatives of PV patients in high-prevalence regions, suggesting that additional triggering factors beyond genetic susceptibility are required for disease expression [3]. Formation of drug-induced neoantigens or stimulation of autoantibody production against desmoglein 3 and/or desmoglein 1 represents the most plausible mechanism underlying drug-induced PV [2,3]. The present case may therefore reflect either an idiopathic process or a "two-hit" phenomenon in which sequential exposures converge to precipitate clinical disease.

In this patient, a predisposing factor was already present in the form of chronic lisinopril use [12]. Upon admission in critical condition requiring vasopressor support, home medications including lisinopril were discontinued. In this case, Lisinopril could not be considered a sole alternative cause but rather a possible immunological primer for the cutaneous presentation, as it had already been discontinued upon admission. IV ceftriaxone was initiated, potentially serving as a second immunologic trigger and precipitating a two-hit effect that led to the clinical manifestation of drug-induced blistering. Patients with prior exposure to pemphigus-associated medications may harbor subclinical immune priming, rendering them susceptible to overt disease upon encountering an additional provocative agent. Reports of ceftriaxone-induced PV remain scarce. A comparable scenario was described in a patient with known HLA predisposition who initially developed PV following exposure to feprazone, a pyrazolone derivative; although symptoms persisted after feprazone withdrawal, they worsened markedly when ceftriaxone was administered for acute pharyngitis [11]. Ruocco et al. have similarly noted that cefixime, another cephalosporin, may trigger or exacerbate PV in genetically predisposed individuals, with characteristic mucocutaneous lesions emerging after drug exposure [6]. The systematic review by Ghaedi et al. confirms that drug-induced PV is clinically and histopathologically indistinguishable from idiopathic disease but may carry a more favorable prognosis when the causative agent is identified and promptly discontinued [2].

Although a definitive causal link cannot always be established, the temporal relationship between ceftriaxone initiation, lesion onset, and clinical improvement following drug withdrawal in this case supports a drug-induced mechanism. Using standardized causality assessment tools, this case meets criteria for “probable” association on the World Health Organization-Uppsala Monitoring Centre (WHO-UMC) scale and “probable” association on the Naranjo adverse drug reaction probability scale (Table 2) [13]. The WHO-UMC system classifies causal association as certain, probable/likely, possible, unlikely, conditional/unclassified, or unassessable/unclassifiable. The "certain" category requires, among other criteria, either a positive rechallenge or definitive pharmacological/phenomenological evidence, and the event must not be explainable by the disease or other drugs. As rechallenge with ceftriaxone would be ethically contraindicated in this clinical context, the highest achievable classification is "probable," which is consistent with the assessment applied in this case.

**Table 2:** Naranjo adverse drug reaction probability scale The Naranjo algorithm is a 10-item questionnaire, each scored as "yes," "no," or "unknown," with a total score categorized as definite (≥9), probable (5-8), possible (1-4), or unlikely (≤0) [13]. Using a standardized causality assessment tool, this case meets criteria for “probable” association on the Naranjo adverse drug reaction probability scale PV: pemphigus vulgaris

Naranjo question	Answer	Score
1. Are there previous conclusive reports of this reaction?	Yes (rare cephalosporin-induced PV reports exist)	+1
2. Did the adverse event appear after the suspected drug was given?	Yes	+2
3. Did the reaction improve when the drug was discontinued?	Yes	+1
4. Did the reaction reappear on rechallenge?	Not done	0
5. Are there alternative causes that could have caused the reaction?	No	+2
6. Did the reaction appear when a placebo was given?	Not done	0
7. Was the drug detected in toxic concentrations?	Unknown	0
8. Was the reaction dose-related?	Unknown	0
9. Did the patient have a similar reaction to the same or similar drug previously?	No	0
10. Was the reaction confirmed by objective evidence (e.g., biopsy)?	Yes (histopathology confirmed PV)	+1
Total		7

Other medications administered during hospitalization, including amiodarone, azithromycin, norepinephrine, propofol, fentanyl, midazolam, and heparin, as well as preadmission amphetamine exposure and home medications other than lisinopril, have not been established as causes of drug-induced PV. However, some of these agents, particularly amiodarone and azithromycin, have been associated with severe cutaneous adverse reactions, including SJS/TEN [14,15]. These diagnoses were excluded in the present case because histopathologic examination did not demonstrate full-thickness epidermal necrosis or subepidermal cleavage, findings characteristic of SJS/TEN [16]. Additionally, low-molecular-weight heparin has been reported to cause bullous hemorrhagic dermatosis, a distinct condition characterized by hemorrhagic bullae and negative direct immunofluorescence findings, which should be differentiated from autoimmune blistering diseases such as PV [17].

The differential diagnosis of intraepidermal blistering includes pemphigus foliaceus, bullous pemphigoid, and paraneoplastic pemphigus. Pemphigus foliaceus typically presents with superficial crusted erosions and subcorneal acantholysis on histology [18]. Bullous pemphigoid is characterized by tense subepidermal blisters with a subepidermal split on biopsy [19]. Paraneoplastic pemphigus, which presents as PV or Pemphigus foliaceus, is associated with underlying malignancy, most commonly non-Hodgkin lymphoma, chronic lymphocytic leukemia, Castleman disease, or thymoma [1,3]. No clinical, hematologic, or radiologic evidence of malignancy was identified in this patient.

Timely recognition of drug-induced PV is essential, as continued exposure to the offending agent can result in widespread mucocutaneous involvement, secondary infection, and sepsis. Prompt drug withdrawal, combined with initiation of immunosuppressive therapy, is the cornerstone of management to reduce morbidity and mortality. In cases refractory to corticosteroids or requiring prolonged treatment, rituximab has demonstrated the ability to induce complete remission off therapy in approximately 90% of patients while permitting rapid corticosteroid tapering, thereby substantially reducing corticosteroid-related adverse events [3]. This case further emphasizes the importance of exercising caution when prescribing broad-spectrum antibiotics empirically in critically ill patients, particularly those with multiple comorbidities and concurrent medication exposures that may predispose to immune dysregulation.

## Conclusions

This case illustrates the potential for ceftriaxone to precipitate PV in genetically or clinically predisposed individuals, despite the extreme rarity of this reaction. The patient's chronic exposure to lisinopril, a known pemphigus-associated medication, may have established a subclinical immunologic predisposition that was subsequently unmasked by ceftriaxone through a plausible two-hit mechanism. The clinical presentation, histopathological findings, and temporal correlation between ceftriaxone initiation and lesion development collectively support a probable drug-induced etiology, while alternative blistering disorders and SJS/TEN were systematically excluded. Clinicians should remain vigilant for autoimmune blistering disorders in patients receiving β-lactam antibiotics and should promptly discontinue the suspected agent when new cutaneous eruptions arise. Early identification, immediate withdrawal of the offending drug, and timely initiation of immunosuppressive therapy are critical to minimizing morbidity and mortality. This report reinforces the need for judicious antibiotic selection and heightened awareness of rare autoimmune cutaneous adverse reactions in critically ill patients with multiple comorbidities and concurrent pharmacologic exposures.
